# Poly[[[μ_3_-5-(pyridine-4-carboxamido)­isophthalato]{μ_3_-5-[(pyridin-1-ium-4-yl)carbonyl­amino]­isophthalato}­neodymium(III)] dihydrate]

**DOI:** 10.1107/S1600536811034532

**Published:** 2011-08-27

**Authors:** Yi-Fang Deng

**Affiliations:** a Department of Chemistry and Materials Science, Hengyang, Hunan 421008, People’s Republic of China

## Abstract

In the title compound, {[Nd(C_14_H_9_N_2_O_5_)(C_14_H_8_N_2_O_5_)]·2H_2_O}_*n*_, the Nd^III^ atom is eight-coordinated as it is surrounded by eight carboxyl­ate O atoms from six ligands in a distorted square-anti­prismatic arrangement. The Nd^III^ atoms are linked by H*L* ^−^ and *L* ^2−^ ligands [H_2_ *L* is 5-(pyridine-4-carboxamido)­isophthalic acid], forming a bilayer network. The layers are linked into a three-demensional network through N—H⋯O and O—H⋯O hydrogen bonds.

## Related literature

For background on transition metal complexes that exhibit one-, two- and three-dimensional frameworks, see: Kitagawa & Kondo (1998 ▶). For high-dimensional lanthanide frameworks, see: Kiritsis *et al.* (1998 ▶); Zhao *et al.* (2004 ▶). For coordination capabilities of carboxyl­ate, pyridine and amide groups, see: Huyskens (1977 ▶); Lee & Kumler (1962 ▶); Wang *et al.* (2007 ▶). 
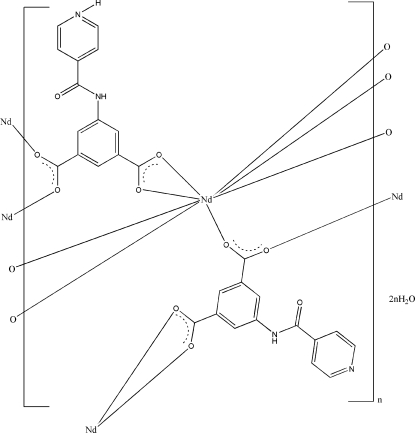


## Experimental

### 

#### Crystal data

[Nd(C_14_H_9_N_2_O_5_)(C_14_H_8_N_2_O_5_)]·2H_2_O
*M* *_r_* = 749.73
Monoclinic, 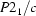

*a* = 13.4421 (15) Å
*b* = 13.7754 (17) Å
*c* = 16.2418 (13) Å
β = 115.813 (4)°
*V* = 2707.4 (5) Å^3^
*Z* = 4
Mo *K*α radiation
μ = 2.00 mm^−1^
*T* = 291 K
0.18 × 0.16 × 0.12 mm

#### Data collection

Bruker SMART APEX diffractometer
Absorption correction: multi-scan (*SADABS*; Sheldrick, 2008 ▶) *T*_min_ = 0.715, *T*_max_ = 0.796
14297 measured reflections
5287 independent reflections
4792 reflections with *I* > 2σ(*I*)
*R*_int_ = 0.052

#### Refinement

*R*[*F* ^2^ > 2σ(*F* ^2^)] = 0.038
*wR*(*F* ^2^) = 0.101
*S* = 1.07
5287 reflections
406 parameters
H-atom parameters constrained
Δρ_max_ = 1.83 e Å^−3^
Δρ_min_ = −1.61 e Å^−3^

### 

Data collection: *SMART* (Bruker, 2001 ▶); cell refinement: *SAINT* (Bruker, 2003 ▶); data reduction: *SAINT*; program(s) used to solve structure: *SHELXS97* (Sheldrick, 2008 ▶); program(s) used to refine structure: *SHELXL97* (Sheldrick, 2008 ▶); molecular graphics: *SHELXTL* (Sheldrick, 2008 ▶); software used to prepare material for publication: *SHELXTL*.

## Supplementary Material

Crystal structure: contains datablock(s) global, I. DOI: 10.1107/S1600536811034532/ng5214sup1.cif

Structure factors: contains datablock(s) I. DOI: 10.1107/S1600536811034532/ng5214Isup2.hkl

Additional supplementary materials:  crystallographic information; 3D view; checkCIF report

## Figures and Tables

**Table 1:** Hydrogen-bond geometry (Å, °)

*D*—H⋯*A*	*D*—H	H⋯*A*	*D*⋯*A*	*D*—H⋯*A*
N2—H2⋯O1*W*	0.86	2.06	2.846 (5)	151
N1—H1⋯O2^i^	0.89	1.85	2.725 (4)	171
O1*W*—H1*WA*⋯O9^i^	0.85	1.89	2.734 (5)	177
O2*W*—H2*WA*⋯O10^i^	0.85	2.52	3.124 (5)	129
O1*W*—H1*WB*⋯O10^ii^	0.85	2.00	2.845 (5)	171
O2*W*—H2*WA*⋯O4^iii^	0.85	2.36	2.923 (5)	125
O2*W*—H2*WB*⋯O1^iv^	0.85	2.11	2.957 (4)	178
N4—H4*A*⋯O2*W*^v^	0.86	2.15	2.953 (5)	156

Symmetry codes: (i) 
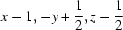
; (ii) 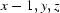; (iii) 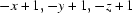; (iv) 
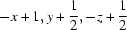
; (v) 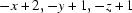.

**Fig. 1.**
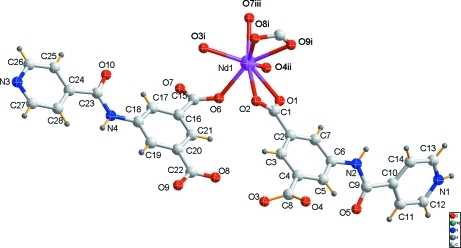
The ORTEP drawing of the title compound (I). Displacement ellipsoids are drawn at 30% probability level. [Symmetry codes: (i) 2 - x,-1/2 + y, 3/2 - z (ii) x,1/2 - y, -1/2 + z (iii) 2 - x,-y, 1 - z.]

**Fig. 2.**
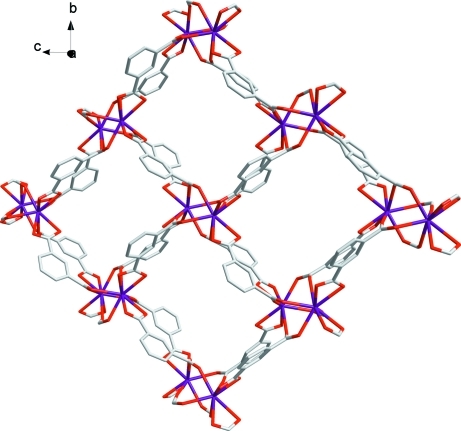
Projection showing the two-dimensional structure of the compound linked by L2-; all the pyridyl groups are omitted.

## supplementary crystallographic information

### Comment

In recent years, there has a great deal of interest in synthesizing transition metal complexes that exhibit one-, two- and three-dimensional frameworks (Kitagawa & Kondo, 1998). However, high-dimensional lanthanide frameworks are less common (Kiritsis *et al.*, 1998; Zhao *et al.*, 2004). On the other hand, it is well known that carboxylate and pyridine groups have good coordination capacities as well as the amide group, a group with two different types of hydrogen bonding sites: the –NH moiety that acts as an electron acceptor and a –C=O group that acts as an electron donor (Lee, & Kumler, 1962; Huyskens, 1977; Wang *et al.*, 2007). The study reports a new lanthanide(III) coordination polymer, [Nd(HL)(*L*)]_n_.2nH_2_O, (I), with H_2_L and Nd(NO_3_)_3_.6H_2_O.

In the title compound, the central Nd^III^ ion is eight-coordinated by eight O atoms from six ligands, which gives a square antiprismatic geometry (Fig. 1). The carboxyl groups of the two unique *L*^2-^ (H*L*^-^) ligands exhibit the same coordination modes: there is a monocarboxylate and a dicarboxylate, i.e., the monocarboxylate group coordinates to one Nd^III^ atom in µ_1_-η^1^:η^1^-chelate mode and the other dicarboxylate connects two Nd^III^ atoms in a µ_2_-η^1^:η^1^ bridging mode. The pyridyl groups are free. Based on the coordination modes of the carboxylate groups of *L*^2-^ (H*L*^-^), a bilayer network is formed (Fig. 2). Adjacent molecules are linked through N—H···O and O—H···O hydrogen bonds into a three-dimensional network.

### Experimental

A mixture of 0.05 mmol Nd(NO_3_)_3_.6H_2_O (21.5 mg. 0.05 mmol), H_2_L (28.6 mg, 0.1 mmol), NaOH (6.0 mg, 0.15 mmol), MeOH (4 ml) and H_2_O (6 ml) was heated in a 16 ml Teflon-lined reaction vessel at 453 K for 5 days; the mixture was cooled to room temperature over a period of 40 h. The product was collected by filtration, washed with H_2_O and air-dried.

### Refinement

H atoms bonded to C atoms were placed geometrically and refiined as riding atoms. The pyridyl (N1) was found from a difference Fourier maps and refined as riding, with N—H = 0.86 Å, and the water H atoms were found from Fourier difference maps and refined with restraints for O—H distances (0.85 Å) with *U*_iso_(H) = 1.2*U*_eq_(O). The highest residual electron density was found at 0.07 Å from Nd1 atom and the deepest hole at 0.56 Å from the O1W atom.

### Crystal data

**Table:**

[Nd(C_14_H_9_N_2_O_5_)(C_14_H_8_N_2_O_5_)]·2H_2_O	*F*(000) = 1492
*M**_r_* = 749.73	*D*_x_ = 1.839 Mg m^−^^3^
Monoclinic, *P*2_1_/*c*	Mo *K*α radiation, λ = 0.71073 Å
Hall symbol: -p 2ybc	Cell parameters from 8058 reflections
*a* = 13.4421 (15) Å	θ = 2.2–28.3°
*b* = 13.7754 (17) Å	µ = 2.00 mm^−^^1^
*c* = 16.2418 (13) Å	*T* = 291 K
β = 115.813 (4)°	Block, colorless
*V* = 2707.4 (5) Å^3^	0.18 × 0.16 × 0.12 mm
*Z* = 4	

### Data collection

**Table:**

Bruker SMART APEX diffractometer	5287 independent reflections
Radiation source: fine-focus sealed tube	4792 reflections with *I* > 2σ(*I*)
graphite	*R*_int_ = 0.052
φ and ω scans	θ_max_ = 26.0°, θ_min_ = 1.7°
Absorption correction: multi-scan (*SADABS*; Sheldrick, 2008)	*h* = −16→15
*T*_min_ = 0.715, *T*_max_ = 0.796	*k* = −16→16
14297 measured reflections	*l* = −20→16

### Refinement

**Table:**

Refinement on *F*^2^	Primary atom site location: structure-invariant direct methods
Least-squares matrix: full	Secondary atom site location: difference Fourier map
*R*[*F*^2^ > 2σ(*F*^2^)] = 0.038	Hydrogen site location: inferred from neighbouring sites
*wR*(*F*^2^) = 0.101	H-atom parameters constrained
*S* = 1.07	*w* = 1/[σ^2^(*F*_o_^2^) + (0.055*P*)^2^ + 3.5595*P*] where *P* = (*F*_o_^2^ + 2*F*_c_^2^)/3
5287 reflections	(Δ/σ)_max_ < 0.001
406 parameters	Δρ_max_ = 1.83 e Å^−^^3^
0 restraints	Δρ_min_ = −1.61 e Å^−^^3^

### Special details

**Table:**

Geometry. All e.s.d.'s (except the e.s.d. in the dihedral angle between two l.s. planes) are estimated using the full covariance matrix. The cell e.s.d.'s are taken into account individually in the estimation of e.s.d.'s in distances, angles and torsion angles; correlations between e.s.d.'s in cell parameters are only used when they are defined by crystal symmetry. An approximate (isotropic) treatment of cell e.s.d.'s is used for estimating e.s.d.'s involving l.s. planes.
Refinement. Refinement of *F*^2^ against ALL reflections. The weighted *R*-factor *wR* and goodness of fit *S* are based on *F*^2^, conventional *R*-factors *R* are based on *F*, with *F* set to zero for negative *F*^2^. The threshold expression of *F*^2^ > σ(*F*^2^) is used only for calculating *R*-factors(gt) *etc*. and is not relevant to the choice of reflections for refinement. *R*-factors based on *F*^2^ are statistically about twice as large as those based on *F*, and *R*- factors based on ALL data will be even larger.

### Fractional atomic coordinates and isotropic or equivalent isotropic displacement parameters (Å^2^)

**Table:**

	*x*	*y*	*z*	*U*_iso_*/*U*_eq_	
O2W	0.4403 (3)	0.6060 (3)	0.0967 (3)	0.0456 (9)	
O1W	0.4253 (3)	0.1460 (3)	0.5360 (2)	0.0537 (11)	
Nd1	0.898403 (16)	0.014994 (16)	0.571291 (14)	0.01719 (10)	
O1	0.7704 (2)	0.1428 (2)	0.56596 (19)	0.0250 (6)	
O2	0.9065 (2)	0.1322 (2)	0.70371 (19)	0.0236 (6)	
O3	0.9144 (2)	0.4679 (2)	0.8775 (2)	0.0289 (7)	
O4	0.7730 (2)	0.4510 (2)	0.91087 (18)	0.0244 (6)	
O5	0.4645 (3)	0.5086 (2)	0.5783 (2)	0.0356 (8)	
O6	0.9932 (2)	0.1532 (2)	0.55514 (19)	0.0212 (6)	
O7	1.1226 (2)	0.1364 (2)	0.50548 (19)	0.0218 (6)	
O8	1.0730 (2)	0.4069 (2)	0.7959 (2)	0.0279 (7)	
O9	1.2447 (2)	0.4326 (2)	0.89481 (19)	0.0255 (7)	
O10	1.4746 (3)	0.0803 (2)	0.7157 (2)	0.0374 (8)	
N2	0.4776 (3)	0.3440 (3)	0.5862 (2)	0.0256 (8)	
H2	0.4395	0.2915	0.5689	0.031*	
N1	0.0898 (3)	0.4082 (3)	0.3610 (3)	0.0343 (9)	
H1	0.0259	0.3971	0.3130	0.041*	
N4	1.4748 (3)	0.2155 (3)	0.7943 (2)	0.0275 (8)	
H4A	1.5175	0.2570	0.8333	0.033*	
N3	1.8768 (3)	0.1047 (4)	0.9233 (4)	0.0524 (13)	
C1	0.8171 (3)	0.1699 (3)	0.6476 (3)	0.0189 (8)	
C2	0.7617 (3)	0.2495 (3)	0.6762 (3)	0.0174 (8)	
C3	0.8178 (3)	0.3080 (3)	0.7518 (3)	0.0177 (8)	
H3	0.8933	0.3002	0.7871	0.021*	
C4	0.7597 (3)	0.3787 (3)	0.7744 (2)	0.0163 (8)	
C5	0.6465 (3)	0.3920 (3)	0.7214 (3)	0.0202 (8)	
H5	0.6080	0.4383	0.7377	0.024*	
C6	0.5922 (3)	0.3350 (3)	0.6440 (3)	0.0197 (8)	
C7	0.6500 (3)	0.2648 (3)	0.6220 (3)	0.0218 (8)	
H7	0.6133	0.2270	0.5700	0.026*	
C8	0.8192 (3)	0.4374 (3)	0.8595 (3)	0.0165 (8)	
C9	0.4240 (3)	0.4284 (3)	0.5564 (3)	0.0240 (9)	
C10	0.3035 (3)	0.4177 (3)	0.4879 (3)	0.0229 (9)	
C11	0.2528 (4)	0.4968 (4)	0.4336 (4)	0.0423 (13)	
H11	0.2917	0.5545	0.4407	0.051*	
C12	0.1446 (5)	0.4904 (4)	0.3688 (4)	0.0490 (15)	
H12	0.1105	0.5430	0.3312	0.059*	
C13	0.1334 (4)	0.3328 (4)	0.4145 (4)	0.0414 (12)	
H13	0.0912	0.2773	0.4082	0.050*	
C14	0.2411 (4)	0.3362 (4)	0.4796 (3)	0.0383 (12)	
H14	0.2717	0.2834	0.5180	0.046*	
C15	1.0895 (3)	0.1640 (3)	0.5638 (3)	0.0178 (8)	
C16	1.1716 (3)	0.2123 (3)	0.6484 (3)	0.0195 (8)	
C17	1.2835 (3)	0.1920 (3)	0.6786 (3)	0.0222 (9)	
H17	1.3067	0.1502	0.6455	0.027*	
C18	1.3594 (3)	0.2342 (3)	0.7580 (3)	0.0254 (9)	
C19	1.3240 (3)	0.2999 (3)	0.8052 (3)	0.0248 (9)	
H19	1.3756	0.3302	0.8576	0.030*	
C20	1.2126 (3)	0.3201 (3)	0.7746 (3)	0.0197 (8)	
C21	1.1354 (3)	0.2748 (3)	0.6964 (3)	0.0212 (8)	
H21	1.0603	0.2863	0.6766	0.025*	
C22	1.1748 (3)	0.3909 (3)	0.8245 (3)	0.0195 (8)	
C23	1.5245 (3)	0.1407 (3)	0.7749 (3)	0.0260 (9)	
C24	1.6479 (3)	0.1320 (3)	0.8288 (3)	0.0244 (9)	
C25	1.7001 (4)	0.0579 (4)	0.8048 (4)	0.0432 (13)	
H25	1.6603	0.0165	0.7563	0.052*	
C26	1.8130 (4)	0.0472 (5)	0.8552 (4)	0.0518 (15)	
H26	1.8468	−0.0042	0.8402	0.062*	
C27	1.8250 (4)	0.1743 (4)	0.9450 (4)	0.0453 (13)	
H27	1.8669	0.2148	0.9935	0.054*	
C28	1.7122 (4)	0.1909 (4)	0.9002 (3)	0.0362 (11)	
H28	1.6804	0.2415	0.9183	0.043*	
H1WA	0.3687	0.1203	0.4936	0.054*	
H1WB	0.4382	0.1202	0.5872	0.054*	
H2WA	0.4156	0.5777	0.1305	0.054*	
H2WB	0.3799	0.6182	0.0501	0.054*	

### Atomic displacement parameters (Å^2^)

**Table:**

	*U*^11^	*U*^22^	*U*^33^	*U*^12^	*U*^13^	*U*^23^
O2W	0.0282 (18)	0.050 (2)	0.050 (2)	0.0082 (17)	0.0092 (16)	0.0003 (19)
O1W	0.056 (2)	0.050 (2)	0.033 (2)	0.025 (2)	−0.0012 (18)	0.0001 (18)
Nd1	0.01348 (13)	0.02044 (15)	0.01550 (14)	0.00045 (7)	0.00432 (10)	0.00084 (8)
O1	0.0206 (14)	0.0288 (16)	0.0190 (14)	0.0067 (12)	0.0026 (12)	−0.0077 (12)
O2	0.0143 (14)	0.0323 (16)	0.0176 (14)	0.0070 (12)	0.0008 (12)	−0.0019 (12)
O3	0.0126 (14)	0.0271 (16)	0.0389 (18)	−0.0022 (12)	0.0037 (13)	−0.0093 (14)
O4	0.0250 (15)	0.0305 (16)	0.0154 (14)	−0.0016 (13)	0.0067 (12)	−0.0050 (12)
O5	0.0210 (17)	0.0343 (19)	0.036 (2)	−0.0009 (13)	−0.0023 (15)	−0.0072 (15)
O6	0.0143 (13)	0.0190 (14)	0.0285 (15)	−0.0013 (11)	0.0076 (12)	−0.0004 (12)
O7	0.0225 (14)	0.0219 (15)	0.0213 (14)	−0.0033 (12)	0.0098 (12)	−0.0039 (12)
O8	0.0141 (14)	0.0380 (18)	0.0269 (16)	0.0020 (13)	0.0046 (12)	−0.0134 (14)
O9	0.0177 (14)	0.0329 (17)	0.0211 (15)	0.0025 (12)	0.0040 (12)	−0.0097 (13)
O10	0.0294 (17)	0.0375 (19)	0.0365 (19)	0.0018 (15)	0.0063 (15)	−0.0118 (16)
N2	0.0120 (16)	0.0273 (19)	0.0258 (19)	−0.0004 (14)	−0.0027 (14)	−0.0088 (15)
N1	0.0175 (18)	0.048 (2)	0.025 (2)	−0.0038 (17)	−0.0017 (16)	0.0043 (18)
N4	0.0122 (16)	0.037 (2)	0.030 (2)	0.0002 (15)	0.0056 (15)	−0.0132 (17)
N3	0.026 (2)	0.051 (3)	0.073 (3)	0.005 (2)	0.015 (2)	−0.008 (3)
C1	0.0180 (19)	0.0190 (19)	0.021 (2)	−0.0013 (16)	0.0092 (17)	−0.0008 (16)
C2	0.0137 (18)	0.0183 (19)	0.0177 (19)	0.0018 (15)	0.0046 (16)	−0.0015 (15)
C3	0.0101 (17)	0.023 (2)	0.0165 (19)	0.0022 (15)	0.0027 (15)	0.0013 (16)
C4	0.0112 (17)	0.0204 (19)	0.0127 (18)	−0.0007 (15)	0.0008 (15)	−0.0021 (15)
C5	0.0152 (19)	0.022 (2)	0.020 (2)	0.0030 (15)	0.0046 (16)	−0.0050 (16)
C6	0.0102 (17)	0.025 (2)	0.0173 (19)	−0.0010 (15)	−0.0002 (15)	−0.0052 (16)
C7	0.0166 (19)	0.025 (2)	0.018 (2)	−0.0007 (16)	0.0023 (16)	−0.0054 (17)
C8	0.0125 (18)	0.0145 (19)	0.0145 (18)	0.0009 (14)	−0.0016 (15)	0.0003 (15)
C9	0.0144 (19)	0.037 (3)	0.017 (2)	0.0015 (18)	0.0030 (16)	−0.0061 (18)
C10	0.0167 (19)	0.031 (2)	0.0173 (19)	0.0016 (17)	0.0040 (16)	−0.0023 (17)
C11	0.026 (3)	0.042 (3)	0.044 (3)	−0.005 (2)	0.002 (2)	0.012 (2)
C12	0.029 (3)	0.056 (4)	0.044 (3)	−0.002 (2)	−0.001 (3)	0.022 (3)
C13	0.023 (2)	0.038 (3)	0.050 (3)	−0.004 (2)	0.003 (2)	0.007 (2)
C14	0.021 (2)	0.040 (3)	0.042 (3)	0.003 (2)	0.002 (2)	0.011 (2)
C15	0.0171 (19)	0.0111 (18)	0.022 (2)	−0.0002 (14)	0.0056 (16)	0.0025 (15)
C16	0.0175 (19)	0.024 (2)	0.0174 (19)	−0.0035 (16)	0.0081 (16)	−0.0018 (16)
C17	0.0186 (19)	0.025 (2)	0.023 (2)	−0.0008 (16)	0.0091 (17)	−0.0073 (17)
C18	0.016 (2)	0.033 (2)	0.025 (2)	0.0006 (17)	0.0076 (17)	−0.0049 (19)
C19	0.018 (2)	0.032 (2)	0.022 (2)	−0.0007 (17)	0.0065 (17)	−0.0077 (18)
C20	0.0191 (19)	0.023 (2)	0.0185 (19)	0.0010 (16)	0.0098 (16)	−0.0014 (16)
C21	0.0139 (18)	0.025 (2)	0.025 (2)	−0.0012 (16)	0.0094 (17)	−0.0026 (17)
C22	0.019 (2)	0.020 (2)	0.021 (2)	−0.0006 (16)	0.0104 (17)	−0.0002 (16)
C23	0.023 (2)	0.029 (2)	0.025 (2)	−0.0020 (18)	0.0097 (19)	−0.0018 (18)
C24	0.017 (2)	0.027 (2)	0.030 (2)	0.0005 (17)	0.0117 (18)	0.0009 (18)
C25	0.032 (3)	0.047 (3)	0.049 (3)	0.002 (2)	0.016 (2)	−0.014 (3)
C26	0.029 (3)	0.055 (3)	0.071 (4)	0.010 (3)	0.021 (3)	−0.018 (3)
C27	0.026 (2)	0.041 (3)	0.055 (3)	−0.003 (2)	0.005 (2)	−0.013 (3)
C28	0.024 (2)	0.033 (3)	0.048 (3)	0.004 (2)	0.013 (2)	−0.009 (2)

### Geometric parameters (Å, °)

**Table:**

O2W—H2WA	0.8499	C2—C3	1.385 (5)
O2W—H2WB	0.8515	C2—C7	1.386 (5)
O1W—H1WA	0.8498	C3—C4	1.393 (5)
O1W—H1WB	0.8499	C3—H3	0.9300
Nd1—O6	2.368 (3)	C4—C5	1.395 (5)
Nd1—O3^i^	2.371 (3)	C4—C8	1.498 (5)
Nd1—O7^ii^	2.383 (3)	C5—C6	1.390 (5)
Nd1—O1	2.436 (3)	C5—H5	0.9300
Nd1—O4^iii^	2.455 (3)	C6—C7	1.381 (5)
Nd1—O9^i^	2.493 (3)	C7—H7	0.9300
Nd1—O8^i^	2.508 (3)	C9—C10	1.521 (5)
Nd1—O2	2.653 (3)	C10—C14	1.373 (6)
O1—C1	1.252 (5)	C10—C11	1.381 (7)
O2—C1	1.261 (5)	C11—C12	1.377 (8)
O3—C8	1.254 (5)	C11—H11	0.9300
O3—Nd1^iv^	2.371 (3)	C12—H12	0.9300
O4—C8	1.252 (5)	C13—C14	1.372 (6)
O4—Nd1^v^	2.455 (3)	C13—H13	0.9300
O5—C9	1.214 (5)	C14—H14	0.9300
O6—C15	1.249 (4)	C15—C16	1.493 (5)
O7—C15	1.267 (5)	C16—C21	1.384 (5)
O7—Nd1^ii^	2.383 (3)	C16—C17	1.392 (5)
O8—C22	1.259 (5)	C17—C18	1.376 (6)
O8—Nd1^iv^	2.508 (3)	C17—H17	0.9300
O9—C22	1.258 (5)	C18—C19	1.397 (6)
O9—Nd1^iv^	2.493 (3)	C19—C20	1.386 (5)
O10—C23	1.226 (5)	C19—H19	0.9300
N2—C9	1.343 (6)	C20—C21	1.389 (6)
N2—C6	1.418 (5)	C20—C22	1.492 (5)
N2—H2	0.8596	C21—H21	0.9300
N1—C13	1.317 (6)	C23—C24	1.505 (6)
N1—C12	1.327 (7)	C24—C28	1.370 (6)
N1—H1	0.8864	C24—C25	1.387 (6)
N4—C23	1.339 (5)	C25—C26	1.383 (7)
N4—C18	1.422 (5)	C25—H25	0.9300
N4—H4A	0.8607	C26—H26	0.9300
N3—C27	1.319 (7)	C27—C28	1.386 (6)
N3—C26	1.326 (7)	C27—H27	0.9300
C1—C2	1.509 (5)	C28—H28	0.9300
			
H2WA—O2W—H2WB	100.1	C7—C6—C5	119.7 (3)
H1WA—O1W—H1WB	110.4	C7—C6—N2	117.5 (3)
O6—Nd1—O3^i^	73.66 (10)	C5—C6—N2	122.7 (4)
O6—Nd1—O7^ii^	126.34 (9)	C6—C7—C2	121.2 (4)
O3^i^—Nd1—O7^ii^	79.05 (10)	C6—C7—H7	119.4
O6—Nd1—O1	79.68 (10)	C2—C7—H7	119.4
O3^i^—Nd1—O1	146.63 (10)	O4—C8—O3	123.2 (4)
O7^ii^—Nd1—O1	133.86 (9)	O4—C8—C4	118.4 (3)
O6—Nd1—O4^iii^	83.28 (10)	O3—C8—C4	118.4 (3)
O3^i^—Nd1—O4^iii^	123.93 (10)	O5—C9—N2	125.5 (4)
O7^ii^—Nd1—O4^iii^	75.02 (10)	O5—C9—C10	120.1 (4)
O1—Nd1—O4^iii^	71.12 (10)	N2—C9—C10	114.4 (4)
O6—Nd1—O9^i^	153.47 (10)	C14—C10—C11	118.2 (4)
O3^i^—Nd1—O9^i^	126.94 (10)	C14—C10—C9	124.2 (4)
O7^ii^—Nd1—O9^i^	77.88 (10)	C11—C10—C9	117.6 (4)
O1—Nd1—O9^i^	74.69 (10)	C12—C11—C10	120.1 (5)
O4^iii^—Nd1—O9^i^	94.70 (9)	C12—C11—H11	120.0
O6—Nd1—O8^i^	133.14 (10)	C10—C11—H11	120.0
O3^i^—Nd1—O8^i^	78.10 (10)	N1—C12—C11	119.0 (5)
O7^ii^—Nd1—O8^i^	82.49 (10)	N1—C12—H12	120.5
O1—Nd1—O8^i^	107.52 (10)	C11—C12—H12	120.5
O4^iii^—Nd1—O8^i^	143.44 (10)	N1—C13—C14	119.9 (5)
O9^i^—Nd1—O8^i^	51.99 (9)	N1—C13—H13	120.1
O6—Nd1—O2	76.49 (9)	C14—C13—H13	120.1
O3^i^—Nd1—O2	102.66 (10)	C13—C14—C10	119.9 (5)
O7^ii^—Nd1—O2	155.57 (9)	C13—C14—H14	120.1
O1—Nd1—O2	50.83 (9)	C10—C14—H14	120.1
O4^iii^—Nd1—O2	120.74 (9)	O6—C15—O7	124.3 (4)
O9^i^—Nd1—O2	82.03 (10)	O6—C15—C16	118.1 (3)
O8^i^—Nd1—O2	74.19 (11)	O7—C15—C16	117.6 (3)
O6—Nd1—C22^i^	152.25 (11)	C21—C16—C17	121.2 (4)
O3^i^—Nd1—C22^i^	102.40 (11)	C21—C16—C15	119.7 (3)
O7^ii^—Nd1—C22^i^	78.11 (10)	C17—C16—C15	119.1 (3)
O1—Nd1—C22^i^	91.90 (11)	C18—C17—C16	119.5 (4)
O4^iii^—Nd1—C22^i^	119.15 (10)	C18—C17—H17	120.3
O9^i^—Nd1—C22^i^	25.98 (10)	C16—C17—H17	120.3
O8^i^—Nd1—C22^i^	26.04 (10)	C17—C18—C19	119.8 (4)
O2—Nd1—C22^i^	77.73 (10)	C17—C18—N4	122.8 (4)
C1—O1—Nd1	99.1 (2)	C19—C18—N4	117.4 (4)
C1—O2—Nd1	88.6 (2)	C20—C19—C18	120.4 (4)
C8—O3—Nd1^iv^	172.9 (3)	C20—C19—H19	119.8
C8—O4—Nd1^v^	115.1 (2)	C18—C19—H19	119.8
C15—O6—Nd1	131.7 (2)	C19—C20—C21	119.9 (4)
C15—O7—Nd1^ii^	130.8 (2)	C19—C20—C22	120.4 (4)
C22—O8—Nd1^iv^	93.0 (2)	C21—C20—C22	119.7 (3)
C22—O9—Nd1^iv^	93.8 (2)	C16—C21—C20	119.2 (4)
C9—N2—C6	124.9 (4)	C16—C21—H21	120.4
C9—N2—H2	117.5	C20—C21—H21	120.4
C6—N2—H2	117.6	O9—C22—O8	121.1 (4)
C13—N1—C12	122.8 (4)	O9—C22—C20	119.7 (3)
C13—N1—H1	115.2	O8—C22—C20	119.1 (4)
C12—N1—H1	121.1	O9—C22—Nd1^iv^	60.3 (2)
C23—N4—C18	127.4 (4)	O8—C22—Nd1^iv^	61.0 (2)
C23—N4—H4A	116.3	O10—C23—N4	123.2 (4)
C18—N4—H4A	116.3	O10—C23—C24	119.3 (4)
C27—N3—C26	115.5 (4)	N4—C23—C24	117.4 (4)
O1—C1—O2	121.5 (4)	C28—C24—C25	117.6 (4)
O1—C1—C2	117.1 (3)	C28—C24—C23	125.0 (4)
O2—C1—C2	121.3 (3)	C25—C24—C23	117.3 (4)
C3—C2—C7	119.7 (4)	C26—C25—C24	118.1 (5)
C3—C2—C1	123.0 (3)	C26—C25—H25	121.0
C7—C2—C1	117.3 (3)	C24—C25—H25	121.0
C2—C3—C4	119.2 (3)	N3—C26—C25	125.1 (5)
C2—C3—H3	120.4	N3—C26—H26	117.5
C4—C3—H3	120.4	C25—C26—H26	117.5
C3—C4—C5	121.0 (3)	N3—C27—C28	124.4 (5)
C3—C4—C8	119.0 (3)	N3—C27—H27	117.8
C5—C4—C8	119.9 (3)	C28—C27—H27	117.8
C6—C5—C4	119.1 (4)	C24—C28—C27	119.3 (4)
C6—C5—H5	120.5	C24—C28—H28	120.4
C4—C5—H5	120.5	C27—C28—H28	120.4
			
O6—Nd1—O1—C1	−81.7 (2)	N2—C9—C10—C14	22.5 (6)
O3^i^—Nd1—O1—C1	−44.5 (3)	O5—C9—C10—C11	18.0 (6)
O7^ii^—Nd1—O1—C1	146.9 (2)	N2—C9—C10—C11	−160.5 (5)
O4^iii^—Nd1—O1—C1	−168.1 (3)	C14—C10—C11—C12	−4.3 (9)
O9^i^—Nd1—O1—C1	91.3 (3)	C9—C10—C11—C12	178.4 (5)
O8^i^—Nd1—O1—C1	50.4 (3)	C13—N1—C12—C11	2.1 (9)
O2—Nd1—O1—C1	−0.8 (2)	C10—C11—C12—N1	1.3 (10)
C22^i^—Nd1—O1—C1	71.7 (3)	C12—N1—C13—C14	−2.4 (9)
O6—Nd1—O2—C1	88.4 (2)	N1—C13—C14—C10	−0.8 (8)
O3^i^—Nd1—O2—C1	157.9 (2)	C11—C10—C14—C13	4.1 (8)
O7^ii^—Nd1—O2—C1	−110.8 (3)	C9—C10—C14—C13	−178.9 (5)
O1—Nd1—O2—C1	0.8 (2)	Nd1—O6—C15—O7	75.6 (5)
O4^iii^—Nd1—O2—C1	14.8 (3)	Nd1—O6—C15—C16	−104.1 (4)
O9^i^—Nd1—O2—C1	−75.9 (2)	Nd1^ii^—O7—C15—O6	−48.7 (5)
O8^i^—Nd1—O2—C1	−128.6 (2)	Nd1^ii^—O7—C15—C16	131.1 (3)
C22^i^—Nd1—O2—C1	−102.0 (2)	O6—C15—C16—C21	−24.5 (5)
O3^i^—Nd1—O6—C15	8.1 (3)	O7—C15—C16—C21	155.8 (4)
O7^ii^—Nd1—O6—C15	−54.4 (4)	O6—C15—C16—C17	154.2 (4)
O1—Nd1—O6—C15	167.8 (4)	O7—C15—C16—C17	−25.5 (5)
O4^iii^—Nd1—O6—C15	−120.2 (3)	C21—C16—C17—C18	0.4 (6)
O9^i^—Nd1—O6—C15	152.7 (3)	C15—C16—C17—C18	−178.3 (4)
O8^i^—Nd1—O6—C15	63.4 (4)	C16—C17—C18—C19	−2.6 (7)
O2—Nd1—O6—C15	115.9 (3)	C16—C17—C18—N4	178.6 (4)
C22^i^—Nd1—O6—C15	93.7 (4)	C23—N4—C18—C17	−18.5 (7)
Nd1—O1—C1—O2	1.5 (4)	C23—N4—C18—C19	162.6 (4)
Nd1—O1—C1—C2	−178.3 (3)	C17—C18—C19—C20	2.4 (7)
Nd1—O2—C1—O1	−1.4 (4)	N4—C18—C19—C20	−178.8 (4)
Nd1—O2—C1—C2	178.5 (3)	C18—C19—C20—C21	0.1 (7)
O1—C1—C2—C3	−158.1 (4)	C18—C19—C20—C22	−179.4 (4)
O2—C1—C2—C3	22.1 (6)	C17—C16—C21—C20	2.0 (6)
O1—C1—C2—C7	20.5 (5)	C15—C16—C21—C20	−179.3 (4)
O2—C1—C2—C7	−159.4 (4)	C19—C20—C21—C16	−2.2 (6)
C7—C2—C3—C4	2.7 (6)	C22—C20—C21—C16	177.3 (4)
C1—C2—C3—C4	−178.8 (4)	Nd1^iv^—O9—C22—O8	4.0 (4)
C2—C3—C4—C5	−0.8 (6)	Nd1^iv^—O9—C22—C20	−175.0 (3)
C2—C3—C4—C8	176.1 (4)	Nd1^iv^—O8—C22—O9	−4.0 (4)
C3—C4—C5—C6	−1.5 (6)	Nd1^iv^—O8—C22—C20	175.1 (3)
C8—C4—C5—C6	−178.3 (4)	C19—C20—C22—O9	0.1 (6)
C4—C5—C6—C7	1.7 (6)	C21—C20—C22—O9	−179.4 (4)
C4—C5—C6—N2	−179.8 (4)	C19—C20—C22—O8	−179.0 (4)
C9—N2—C6—C7	−136.0 (4)	C21—C20—C22—O8	1.5 (6)
C9—N2—C6—C5	45.4 (6)	C18—N4—C23—O10	5.7 (7)
C5—C6—C7—C2	0.3 (6)	C18—N4—C23—C24	−174.7 (4)
N2—C6—C7—C2	−178.3 (4)	O10—C23—C24—C28	−175.8 (5)
C3—C2—C7—C6	−2.5 (6)	N4—C23—C24—C28	4.6 (7)
C1—C2—C7—C6	178.9 (4)	O10—C23—C24—C25	3.3 (6)
Nd1^v^—O4—C8—O3	−22.1 (5)	N4—C23—C24—C25	−176.3 (4)
Nd1^v^—O4—C8—C4	155.6 (3)	C28—C24—C25—C26	0.9 (8)
C3—C4—C8—O4	−135.3 (4)	C23—C24—C25—C26	−178.3 (5)
C5—C4—C8—O4	41.6 (5)	C27—N3—C26—C25	2.8 (10)
C3—C4—C8—O3	42.5 (5)	C24—C25—C26—N3	−2.4 (10)
C5—C4—C8—O3	−140.6 (4)	C26—N3—C27—C28	−1.8 (9)
C6—N2—C9—O5	−4.0 (7)	C25—C24—C28—C27	−0.1 (7)
C6—N2—C9—C10	174.4 (4)	C23—C24—C28—C27	179.1 (5)
O5—C9—C10—C14	−159.0 (5)	N3—C27—C28—C24	0.5 (9)

Symmetry codes: (i) −*x*+2, *y*−1/2, −*z*+3/2; (ii) −*x*+2, −*y*, −*z*+1; (iii) *x*, −*y*+1/2, *z*−1/2; (iv) −*x*+2, *y*+1/2, −*z*+3/2; (v) *x*, −*y*+1/2, *z*+1/2.

### Hydrogen-bond geometry (Å, °)

**Table:**

*D*—H···*A*	*D*—H	H···*A*	*D*···*A*	*D*—H···*A*
N2—H2···O1W	0.86	2.06	2.846 (5)	151.
N1—H1···O2^vi^	0.89	1.85	2.725 (4)	171.
O1W—H1WA···O9^vi^	0.85	1.89	2.734 (5)	177.
O2W—H2WA···O10^vi^	0.85	2.52	3.124 (5)	129.
O1W—H1WB···O10^vii^	0.85	2.00	2.845 (5)	171.
O2W—H2WA···O4^viii^	0.85	2.36	2.923 (5)	125.
O2W—H2WB···O1^ix^	0.85	2.11	2.957 (4)	178.
N4—H4A···O2W^x^	0.86	2.15	2.953 (5)	156.

Symmetry codes: (vi) *x*−1, −*y*+1/2, *z*−1/2; (vii) *x*−1, *y*, *z*; (viii) −*x*+1, −*y*+1, −*z*+1; (ix) −*x*+1, *y*+1/2, −*z*+1/2; (x) −*x*+2, −*y*+1, −*z*+1.
